# Corneal Thickness Response after Anesthetic Eye Drops: Our Own Results and Meta-Analysis

**DOI:** 10.1155/2018/4743721

**Published:** 2018-03-05

**Authors:** Marcelino Perez-Bermejo, Alejandro Cervino, Ana M. Calvo-Maroto, Monica Moscardo, Mayte Murillo-Llorente, Juan A. Sanchis-Gimeno

**Affiliations:** ^1^Catholic University San Vicente Martir, C/Espartero 7, 46007 Valencia, Spain; ^2^Optometry Research Group, Department of Optics, University of Valencia, C/Dr. Moliner 50, Burjassot, 46100 Valencia, Spain; ^3^Ocular Anatomy Unit, Department of Anatomy and Human Embryology, University of Valencia, Faculty of Medicine, Avda. Blasco Ibanez 15, 46010 Valencia, Spain

## Abstract

We aimed to test if there are different patterns in the central corneal thickness (CCT) response after instilling oxybuprocaine anesthetic eye drops and also to determine whether there is a significant change in the CCT. CCT was measured in 60 eyes of 60 healthy subjects before and during the hour after oxybuprocaine 0.4% eye drops were instilled. In addition, a systematic review and meta-analysis were carried out in order to answer the following PICO (patient, intervention, comparison, and outcome) question: What effect do anesthetic eye drops have on CCT values? We found no significant changes in the mean CCT values during the hour's observation (ANOVA, *p* = 0.209), and the meta-analysis revealed no statistically significant changes in the CCT after anesthesia (*Q*-Value = 1.111; *p* value = 1.000; *I*2 = 0.000; Tau2 = 0.000; Stderr = 0.020). However, we found three CCT response patterns 5 minutes after anesthesia: Pattern 1, subjects with no significant changes in their CCT values (*n* = 14, 46.7%); Pattern 2, subjects with significant CCT increases (*n* = 11, 36.7%); and Pattern 3, subjects with significant CCT decreases (*n* = 5, 16.7%). In sum, there are no significant changes in the CCT after anesthesia, but there are three different CCT response patterns 5 minutes after anesthesia.

## 1. Introduction

Central corneal thickness (CCT) assessment is very important in clinical practice as it is a sensitive indicator of corneal health and physiological performance [1]. Accurate assessment of CCT is important in many clinical situations, such as the diagnosis of corneal ectasia conditions and corneal physiology, contact lens research, and monitoring progression of various corneal pathologies, and as an aid in preoperative evaluation for patients undergoing corneal surgery [2–5]. In addition, CCT monitoring for each patient is necessary in up-to-date glaucoma management as there is a statistically significant association between CCT and intraocular pressure (IOP) [6]. In fact, it has been stated that CCT measurement should be the first step in diagnosing IOP pathologies [7].

Conventional ultrasound pachymetry has been considered the gold standard technique for CCT measurement over the last decades [1, 4, 8] but it requires the instillation of anesthetic eye drops. Furthermore, noncontact optical methods, like noncontact scanning-slit corneal topography, make it possible to measure CCT before and after the instillation of anesthetic eye drops [5], the difference between the pre- and postanesthetic CCT values being a consequence of the anesthetic eye drops used [5].

Some authors have studied the effect of anesthetic eye drops on the CCT and have found significant changes after anesthesia [5, 9, 10] while other authors found no significant differences between the pre- and postanesthetic CCT values [3, 11–14]. However, it seems that some subjects can present CCT differences between pre- and postanesthesia with oxybuprocaine eye drops that can range from −30 to +30 *μ*m [3, 5, 11].

In the light of these individual variations in the CCT after oxybuprocaine eye drops are instilled, we aimed to determine whether there are different CCT response patterns after anesthesia with oxybuprocaine eye drops. In addition, we aimed to analyze the CCT changes in the hour after oxybuprocaine eye drop instillation.

## 2. Materials and Methods

### 2.1. Subjects

We carried out a prospective comparative study in healthy emmetropic subjects. Ethics approval from the Ethics Committee of the University of Valencia was obtained prior to conducting the study. The work was performed in accordance with the World Medical Association's Declaration of Helsinki and written informed consent was obtained from all patients.

We recruited 183 volunteers who agreed to participate in the study after the study protocol and the procedures to be carried out had been explained to them.

All the subjects were summoned to an ophthalmologic examination so that the exclusion and inclusion criteria could be applied. Exclusion criteria were prior corneal and/or ocular surgery, corneal disease, clinical corneal changes, and Goldmann applanation tonometry ≥ 21 mm Hg. Patients with a systemic disease and best-corrected visual acuity < 20/20 and those taking any kind of medication were also excluded [3, 5]. Inclusion criteria were emmetropic subjects (volunteers with manifest sphere and manifest cylinder of ±0.5 diopters), with best-corrected visual acuity ≥ 20/20, and Goldmann applanation tonometry ≤ 20 mmHg [3, 5].

Forty-five subjects from the initial sample of 183 (24.6%) did not keep their appointment for the ophthalmologic examination and were excluded from the study.

After we applied the inclusion and exclusion criteria, we found that 65 of the initial 183 volunteers (35.5%) were healthy emmetropic subjects. Finally, the corneal thickness measurements were carried out in 60 of the 65 emmetropic subjects (92.3%) as five of them (7.7%) abandoned the study before their CCT measurements were carried out. As a result, the CCT measurements were carried out in 60 (32.8%) of the initial 183 volunteers.

### 2.2. CCT Measurements

The CCT measurements were determined by means of noncontact scanning-slit corneal topography (Orbscan topography system II, Orbscan, Inc., Salt Lake City, UT, USA) following literature procedures [3, 5]. The mean of five consecutive CCT measurements was obtained at the central cornea. The acoustic equivalent correction factor of 0.92 recommended by the manufacturer was not used, as suggested in the literature [15]. All CCT measurements were determined between 10 a.m. and 11 a.m. The temperature during the CCT measurement ranged from 18° to 22°C and the relative humidity ranged from 38% to 45%.

The CCT measurements were carried out as follows: baseline measurements were determined by one physician three minutes after two saline solution eye drops were instilled in the eyes of the volunteers. Then either two anesthetic eye drops or two saline solution eye drops were instilled randomly in the eyes of the volunteers. Another physician, who was not aware of the baseline results obtained, measured the CCT again five minutes, 15 minutes, 30 minutes, and 60 minutes after anesthetic eye drops or saline solution eye drop instillation. Each patient was asked to blink before CCT measurement to avoid any bias due to corneal drying. The second physician was not aware of whether saline solution eye drops or anesthetic eye drops had been instilled.

The case group was composed of subjects who were anesthetized while the control group comprised subjects who had not received corneal anesthesia. Demographic characteristics of both groups are presented in Table 1.

### 2.3. Anesthetic Eye Drops

Oxybuprocaine CLH 0.4% eye drops were instilled in the eyes of the volunteers. The eye drops contained thimerosal, boric acid, and purified water as preservatives.

### 2.4. Systematic Review and Meta-Analysis

The review text was structured in accordance with guidelines from PRISMA (Preferred Reporting Items for Systematic Reviews and Meta-Analyses) [16].

The aim of this review was to obtain the answer to the following PICO (patient, intervention, comparison, and outcome) question [17]: What effect do anesthetic eye drops have on CCT values?

An electronic literature search was conducted in several databases, including MEDLINE, EMBASE, and COCHRANE, and addressed articles published between January 1992 and June 2017 in the English language. MESH (for Medical Subject Headings) terms, keywords, and other free terms and Boolean operators (AND, OR) were used to combine searches based on the search strategy previously reported.

For the screening process in databases, the following terms were used: “topical anesthesia and corneal thickness”, “oxybuprocaine and corneal thickness”, “proparacaine and corneal thickness”, “tetracaine and corneal thickness”, “benoxinate and corneal thickness”, and “anesthetic eye drops and corneal thickness”. Two reviewers independently obtained the data from the studies (MM and MP-B). In the case of disagreement, consensus was reached by discussion with a third reviewer (JAS-G).

Articles were included in the systematic review if they met the following inclusion criteria: (1) articles published in Journals included in the Journal Citation Report Science Edition; (2) human study; (3) prospective; (4) studies that reported CCT values before and after oxybuprocaine eye drop instillation. Articles excluded from the systematic review were (1) animal studies; (2) case reports; (3) reviews; (4) studies that did not fulfill the above inclusion criteria; and (5) studies that reported information that was not clear enough or was inconsistent. The quality of such studies was assessed by two masked examiners using the Newcastle-Ottawa scale (NOS) [18]. The NOS assigns a maximum of nine stars; a minimum of six were required to be included in our study.

### 2.5. Statistical Analysis

We analyzed only one eye per patient with a view to eliminating the possible intrasubject effect that would appear if both eyes of the same patient were studied [19]. Only the left eye was contemplated for statistical analysis. The choice of limiting the study to the left eye instead of the right was random. The Kolmogorov-Smirnov test and Student's *t*-test, Man–Whitney *U* test, ANOVA, and Pearson's correlation coefficient were applied. *p* values of less than 0.05 were considered to be statistically significant. All these statistical analyses were carried out using SPSS software (version 19, SPSS, Inc.).

In the meta-analysis and for the outcomes based on continuous data, mean difference (MD) and standard mean difference (SMD) were used. Subsequently, 95% confidence intervals (CIs) and *p* values were calculated using comprehensive meta-analysis software [20] in all outcomes. Statistical heterogeneity was assessed based on the value of *p* and *I*^2^ using the standard *χ*2 test. When *I*^2^ > 50% and *p*  < 0 .1 were considered to be of significant heterogeneity, a random-effect model was performed for meta-analysis. Otherwise, the fixed-effect model was used. Publication bias was evaluated qualitatively by observing asymmetry of funnel plots. Therefore, our meta-analysis has no publication bias. *p* ≤ 0.05 was considered to indicate a statistically significant difference.

## 3. Results

Table 2 shows the CCT values obtained during the study in the case and the control groups. No significant differences were found between the groups.

No significant changes in the CCT values were observed during the hour's analysis in the case group (ANOVA, *p* = 0.209) nor in those of the control group (ANOVA, *p* = 0.814).

Pearson's correlation coefficient found no correlation between the age and the CCT before anesthesia (*r* = −0.160, *p* = 0.400), nor 5 minutes (*r* = −0.125, *p* = 0.510), 15 minutes (*r* = −0.129, *p* = 0.495), 30 minutes (*r* = −0.153, *p* = 0.418), or 60 minutes (*r* = −0.162, *p* = 0.393) after anesthesia, neither was any correlation found between the tonometry and the CCT before anesthesia (*r* = 0.217, *p* = 0.249), or 5 minutes (*r* = 0.216, *p* = 0.251), 15 minutes (*r* = 0.228, *p* = 0.226), 30 minutes (*r* = 0.224, *p* = 0.234), or 60 minutes (*r* = 0.207, *p* = 0.273) after anesthesia.

In the control group, Pearson's correlation coefficient found no correlation between age and the CCT before saline solution instillation (*r* = −0.187, *p* = 0.323), or 5 minutes (*r* = −0.186, *p* = 0.324), 15 minutes (*r* = −0.175, *p* = 0.354), 30 minutes (*r* = −0.174, *p* = 0.358), or 60 minutes (*r* = −0.167, *p* = 0.376) after saline solution instillation, neither was any correlation found between the tonometry and the CCT before saline solution instillation (*r* = 0.129, *p* = 0.498), or 5 minutes (*r* = 0.129, *p* = 0.496), 15 minutes (*r* = 0.124, *p* = 0.513), 30 minutes (*r* = 0.115, *p* = 0.545), or 60 minutes (*r* = 0.109, *p* = 0.566) after saline solution.

However, we found 16 subjects (53.3%) in the case group who presented increases and decreases in their CCT values of over 10 *μ*m 5 minutes after anesthesia. The mean changes in CCT values 5 minutes after anesthesia ranged from −30 *μ*m to +31 *μ*m (mean ± SD, −7.38 ± 19 *μ*m) in those 16 subjects while it ranged from −9 *μ*m to +9 *μ*m (mean ± SD, 1.6 ± 6 *μ*m) in the other 14 subjects.

Table 3 presents the comparison between the subjects who presented changes in CCT values < 10 *μ*m and > 10 *μ*m 5 minutes after anesthesia. No significant differences were found between those subgroups of subjects except for the CCT basal value although the *p* value was almost nonsignificant (*p* = 0.049).

**Figure 1:**
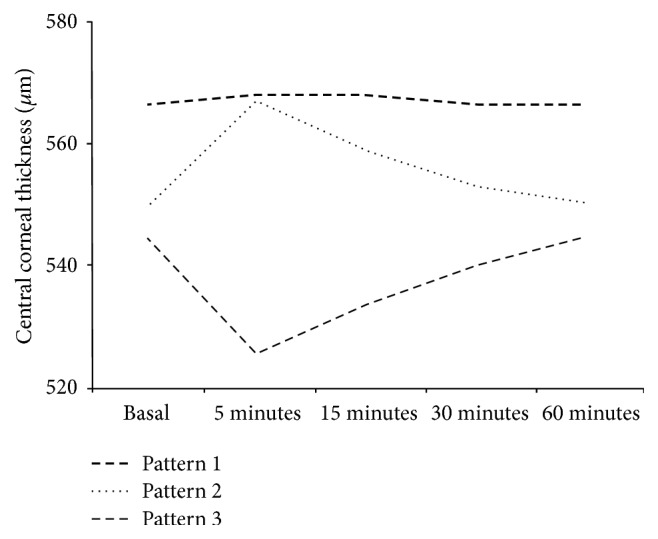
There are three different patterns after oxybuprocaine 0.4% eye drop instillation: Pattern 1, subjects with no significant changes in their CCT values 5 minutes after anesthesia; Pattern 2, subjects with significant CCT increases 5 minutes after anesthesia; and Pattern 3, subjects with significant CCT decreases 5 minutes after anesthesia.

In-depth analysis of the results presented in Table 4 revealed that the changes in the CCT values were significant in the subgroup of volunteers who presented CCT increases >10 *μ*m 5 minutes after anesthesia. The same occurred in the subjects who presented CCT increases <10 *μ*m 5 minutes after anesthesia. Thus, three different patterns could be observed after anesthesia (Figure 1): Pattern 1, subjects with no significant changes in their CCT values 5 minutes after anesthesia (*n* = 14, 46.7%); Pattern 2, subjects with significant CCT increases 5 minutes after anesthesia (*n* = 11, 36.7%); and Pattern 3, subjects with significant CCT decreases 5 minutes after anesthesia (*n* = 5, 16.7%).

In the subgroup of subjects who presented changes in CCT values < 10 *μ*m 5 minutes after anesthesia, the differences between the basal CCT values and those obtained after 15, 30, and 60 minutes were +1.4 ± 5 *μ*m (range from −8 *μ*m to +8 *μ*m), +0.1 ± 2.2 *μ*m (range from −5 *μ*m to +3 *μ*m), and −0.3 ± 0.7 *μ*m (range from −2 *μ*m to +1 *μ*m), respectively. In the subgroup of subjects who presented increases in CCT values > 10 *μ*m 5 minutes after anesthesia, the differences between the basal CCT values and those obtained after 15, 30, and 60 minutes were +9.4 ± 2.4 *μ*m (range from +5 *μ*m to +12 *μ*m), +3.4 ± 1.4 *μ*m (range from +1 *μ*m to +5 *μ*m), and +0.8 ± 0.7 *μ*m (range from 0 *μ*m to +2 *μ*m), respectively. Finally, in the subgroup of subjects who presented decreases in CCT values > 10 *μ*m 5 minutes after anesthesia, the differences between the basal CCT values and those obtained after 15, 30, and 60 minutes were −11 ± 3.7 *μ*m (range from −5 *μ*m to −16 *μ*m), −4.4 ± 1.4 *μ*m (range from −2 *μ*m to −6 *μ*m), and 0 ± 0.6 *μ*m (range from −1 *μ*m to +1 *μ*m), respectively.

**Figure 2:**
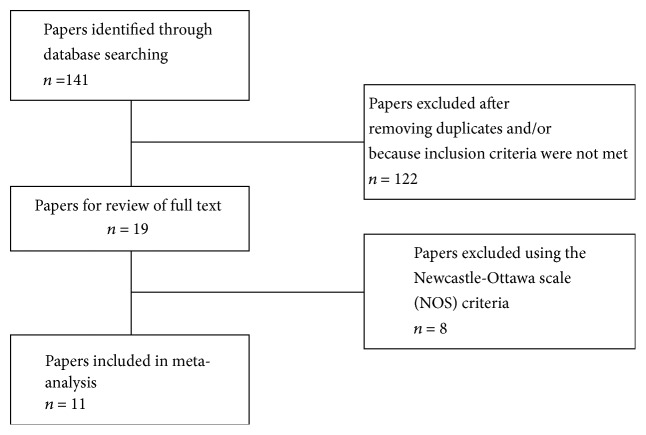
Flowchart of the search process.

The selection process of the studies for the systematic review and the meta-analysis is shown in Figure 2. A total of 141 studies were addressed from the databases. Of these, 122 were excluded. As a result, 19 articles were reviewed and finally eleven were included in the meta-analysis. The characteristics of the eleven studies included in the meta-analysis are summarized in Table 5.

**Figure 3:**
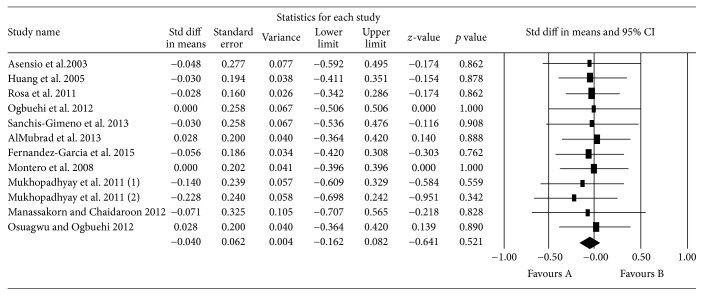
*Q*-Value= 1.111; *p* value = 1.000; *I*2 = 0.000; Tau2 = 0.000; Stderr = 0.020. Mukhopadhyay et al., 2011 (1) = Orbscan IIz measurements; Mukhopadhyay et al., 2011 (2) = Pentacam measurements.

**Figure 4:**
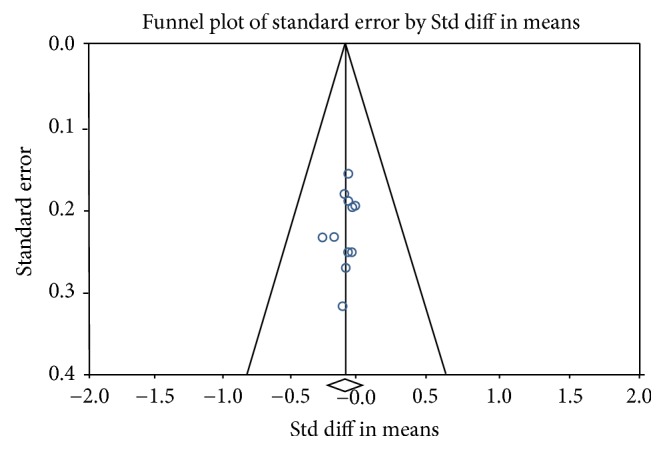
High symmetry that implies that there is no publication bias in the studies of this meta-analysis.

Figure 3 presents the results of the meta-analysis. The model, although not significant (*p* = 1,000), shows a standardized difference of 0.024, a very low value that shows that there are no differences between the CCT values before and after anesthesia. The value of *I*^2^ indicates that there is no evidence of heterogeneity in the studies (Figure 4).

## 4. Discussion

The present study analyzes the CCT changes during the hour after the cornea was anesthetized with oxybuprocaine 0.4% eye drops, which are used in visual examinations like applanation tonometry, ultrasonic pachymetry, ocular biometry, and so on. Moreover, CCT measurements were performed in a single blinded manner by two investigators. Only emmetropic eyes were involved in this study because they are considered to be “normal” from the anatomical point of view [26].

In this study, we observed that CCT values after instillation of anesthetic drops were not statistically different compared to those CCT values before instillation. However, we observed three different response patterns 5 minutes after instilling drops (significant increase, significant decrease, and no significant changes). This fact has not been described in the literature to date.

Oxybuprocaine CLH 0.4% eye drops were used in this study. Oxybuprocaine is also known as benoxinate [4-amino-3-butoxybenzoic acid-2-(diethylamino)-ethyl ester], which is an agent with an ester, and it is available as a 0.4% solution. These drops act as an anesthetic by sodium channels blockage, located at nerve endings of the cornea. Instillation of oxybuprocaine 0.4% eye drops causes full anesthesia within 60 seconds [11]; however basal sensitivity levels recover only after 60 minutes [27].

Moreover, local anesthetics can modify Na+/K+ ATPase activity on corneal endothelium and corneal osmotic pressure, and consequently corneal stroma hydration increases [11, 28]. Therefore, adverse pharmacological effects associated with local anesthetics could explain the increase in CCT values after anesthesia instillation, mainly because corneal changes can even be caused when anesthesia is instilled in small concentrations [29].

In this study, oxybuprocaine drops included thimerosal 0.1 mg/ml, 1 mg of boric acid, and purified water as preservatives. Thimerosal is an organomercury compound that contains 49% mercury used to prevent bacterial contamination, especially* Pseudomonas aeruginosa*, and it is also an antifungal agent. Several authors showed that organomercury compounds can alter membrane permeability and transport systems [30], while others demonstrated retraction of epithelial cells and cancellation of corneal and epithelial cell mitotic activity [31]. Therefore, an increase in CCT values after anesthetic eye drop instillation could be caused by the potential toxicity of thimerosal. Furthermore, postanesthetic corneal edema might explain this increase in CCT values after anesthetic eye drops have been administered [9]. This fact could be related to tearing when anesthetic eye drops are instilled, which is not desirable due to the lineal relation between CCT and corneal hydration [32]. In addition, local anesthetics and preservatives can cause toxicity in the corneal surface [29, 31, 33–36]. Thus, instillation of these drops could alter corneal epithelium properties and impair the barrier function leading to a change in corneal hydration, and hence CCT values.

Previous studies showed that oxybuprocaine HCL (0.4%) did not cause statistically significant changes in the CCT mean value after eye drop instillation [3, 11–14, 21]. Ogbuehi [37] evaluated CCT values from 26 eyes by specular microscopy 5 minutes after oxybuprocaine HCL drop instillation and observed that CCT changes were not statistically significant. Almubrad et al. [14] observed that CCT changes were not statistically significant either in 50 eyes studied by SM 5 minutes after instillation.

However, Fernandez-Garcia et al. [5] analyzed 58 eyes from 58 patients (30 women and 28 men) by Orbscan pachymetry 3 minutes after oxybuprocaine eye drop instillation. They observed that women showed a statistically significant CCT increase, which was located at the bottom of the corneal zone. This occurrence was not observed in men. The authors associated this observation with estrogen influence that can cause changes in corneal hydration and CCT values. This possible association between hormones and corneal physiology in women is strengthened during pregnancy and the menstrual cycle [5, 38, 39]. Nam et al. [10] analyzed 18 eyes from 18 subjects by SM and compared CCT values after instilling one drop and two drops of oxybuprocaine HCL (0.4%), respectively. Both situations showed a transient increase in CCT, but it was not statistically significant.

Huang et al. [21] analyzed when Orbscan II pachymetry should be conducted, before or after Goldmann applanation tonometry. They used oxybuprocaine eye drops. They evaluated CCT values after drop instillation, and they concluded that the use of anesthetic eye drops did not modify CCT values measured by the Goldmann applanation tonometry test.

As mentioned above, several studies conclude that CCT changes after anesthetic instillation are not statistically significant. However, when these changes were analyzed in each subject, it has been observed that this (transient) increase in CCT values was statistically significant. Rosa et al. [12] analyzed 78 eyes from 78 subjects and found statistically significant changes 5 minutes after eye drop instillation. Moreover, one patient showed an increase of up to 20 *μ*m in the CCT measurement 5 minutes after instillation. In addition, Asensio et al. [11] studied the effect of oxybuprocaine on CCT measurements in 26 eyes from 26 patients and they observed that several patients showed an increase or decrease of up to 14 *μ*m, 3 minutes after anesthetic instillation. The authors concluded that these increases and decreases in CCT measurements could be due to edema or corneal peeling after anesthetic instillation.

With regard to proparacaine 0.5%, CCT changes after drop instillation have been observed [9, 10].

In a study where 18 eyes from 18 subjects were analyzed by specular microscopy, the authors observed that the highest increase in the CCT value was 8.6 *μ*m 20 seconds after drop instillation, and, subsequently, it returned to its basal value. Another increase of 6.8 *μ*m was observed 4 minutes and 40 seconds after instillation. This last increase demonstrated that the CCT value was unstable around 5 minutes after anesthetic instillation [10]. Tear film instability or the appearance of corneal edema in response to preservatives can explain this increase. Proparacaine 0.5% contains benzalkonium chloride 0.01%, while the preservatives in oxybuprocaine are thimerosal, sodium chloride, edetate disodium, boric acid, and purified water. However, the effect of these preservatives on tear film and CCT values requires further research [10], although ophthalmic solutions that have benzalkonium chloride as a preservative have been observed to reduce the noninvasive break-up time (NIBUT) in human eyes [40].

Mukhopadhyay et al. [22] evaluated CCT measurements from 35 eyes after eye drop instillation composed of proparacaine 0.5% and fluorescein sodium 0.25%. They analyzed the CCT changes in each subject and observed that CCT measurements can decrease by 10 *μ*m or increase up to 30 *μ*m. These results are consistent with those obtained by Asensio et al. [11] who used oxybuprocaine.

Nonetheless, other authors observed that CCT changes caused by proparacaine 0.5% were not statistically significant [9, 41]. Even so, Herse and Siu [9] observed that CCT measurements increased significantly 1 or 2 minutes after instilling 2 drops in 10 eyes. They attributed these changes to transient corneal edema formation [9].

Regarding tetracaine hydrochloride 0.5%, Manassakorn and Chaidaroon [23] observed that CCT changes were not statistically significant after drop instillation in 19 eyes with glaucoma. They measured it at 2-minute intervals for 15 minutes after eye drop instillation and observed how these measurements reached a peak 1 minute after drop instillation, but it was not statistically significant compared to basal values. Ogbuehi et al. [13] evaluated CCT values from 30 eyes by SM 5 minutes after tetracaine hydrochloride 0.5% instillation and they observed that the changes were not statistically significant. In addition, Osuagwu and Ogbuehi [24] analyzed CCT measurements by SM after instilling one drop of tetracaine HCL 1% in 50 subjects. They observed that these variations were not statistically significant.

The combination of tetracaine HCL 0.1% and oxybuprocaine HCL 0.4% has also been studied by Díaz-Rey et al. [42]. They evaluated CCT values from 12 eyes by Orbscan II pachymetry 4 minutes after drop instillation and the differences were not statistically significant. Montero et al. [25] found no statistically significant differences in their study, in which they evaluated CCT values from 80 eyes by Orbscan II pachymetry 5 minutes after instillation of this combination.

## 5. Conclusions

In conclusion, the results of meta-analysis show that eye drop instillation of local anesthesia on the ocular surface causes variations in the CCT mean value, but it is not statistically significant. However, the scientific literature indicates that CCT values can increase or decrease significantly in certain cases. Additionally, a bibliographic review reveals that eye drop instillation of anesthesia might have greater effects on CCT measurements in women due to the relationship between estrogen and CCT values. Furthermore, certain subjects undergo a decrease or increase in CCT measurements of up to 30 *μ*m after eye drop instillation. This interindividual variability should be considered in the interpretation of CCT measurements by contact measuring methods like ultrasonic pachymetry or after applanation tonometry. Due to the effect of anesthetic drop instillation on CCT measurements, it would be appropriate to use noncontact methods to avoid the need of instilling anesthetic drops.

According to our experimental results, we conclude that there are three different response patterns to CCT measurements 5 minutes after instillation of oxybuprocaine drops (a significant increase, a significant decrease, and no significant changes) which clinicians should be aware of when analyzing CCT measurements after anesthetic drop instillation.

## Figures and Tables

**Table 1:** Demographic characteristics of case and control group subjects.

	Case group (*n* = 30)	Control group (*n* = 30)	*p* value^*∗*^
	Mean ± SD	Mean ± SD	
Age (years)	28.3 ± 6.2	27.9 ± 4.5	0.776
MSE (diopters)^†^	−0.1 ± 0.4	0.0 ± 0.3	0.411
Tonometry (mmHg)	15.5 ± 1.2	15.9 ± 1.3	0.375

^*∗*^Student's *t*-test; ^†^manifest spherical equivalent; BCVA: best corrected visual acuity.

**Table 2:** Central corneal thickness values obtained in case and control group subjects (*µ*m).

	Case group (*n* = 30)		Control group (*n* = 30)		*p* value^*∗*^
	Mean ± SD	Range	Mean ± SD	Range	
Baseline CCT	558 ± 19	525–592	556 ± 15	535–584	0.684
CCT 5 minutes	563 ± 24	500–599	556 ± 15	535–584	0.223
CCT 15 minutes	562 ± 21	515–595	556 ± 15	535–584	0.264
CCT 30 minutes	559 ± 19	521–588	556 ± 15	535–584	0.513
CCT 60 minutes	558 ± 19	526–590	556 ± 15	535–584	0.658

^*∗*^Student's *t*-test; CCT: central corneal thickness values.

**Table 3:** Characteristics of the subjects who presented CCT value changes < 10 *µ*m and >10 *µ*m 5 minutes after anesthesia instillation.

	CCT changes < 10 *µ*m	CCT changes > 10 *µ*m	*p* value^*∗*^
*n*	14 (46.7%)	16 (53.3%)	---
Age (years)	29.3 ± 7.5	27.4 ± 4.8	0.422
MSE (diopters)	−0.2 ± 0.3	0.0 ± 0.4	0.358
Tonometry (mmHg)	15.9 ± 1.6	15.2 ± 1.4	0.196
CCT basal (*µ*m)	566 ± 19	552 ± 19	0.049^†^
CCT 5 minutes (*µ*m)	568 ± 17	560 ± 30	0.353
*p* value	0.337	0.149	---

^*∗*^Student's *t*-test; ^†^statistically significant; CCT: central corneal thickness; MSE: manifest spherical equivalent.

**Table 4:** Comparison between the subjects who showed CCT increases and decreases >10 *µ*m 5 minutes after anesthesia instillation.

	Increases > 10 *µ*m	Decreases > 10 *µ*m	*p* value^*∗*^
*n*	11 (36.7%)	5 (16.7%)	---
Age (years)	27.3 ± 5.1	27.8 ± 4.5	0.841
MSE (diopters)	−0.3 ± 0.2	0,0 ± 0.3	0.126
Tonometry (mmHg)	15.5 ± 1.4	14.6 ± 1.7	0.353
CCT basal (*µ*m)	555 ± 15	545 ± 25	0.409
CCT 5 minutes (*µ*m)	575 ± 12	526 ± 30	0.020^†^
*p* value	<0.001^†^	0.009^†^	---

^*∗*^ *U* Mann–Whitney; ^†^Statistically significant; CCT: central corneal thickness; MSE: manifest spherical equivalent.

**Table 5:** Articles included in the meta-analysis.

Authors	*n*	Anesthetic	CCT before anesthesia (*µ*m)	CCT after anesthesia (*µ*m)	Minutes after anesthesia	Pachymetry
Sanchis-Gimeno et al. [3]	30	Oxybuprocaine hcl 0.4%	599 ± 33	600 ± 34	3	Orbscan II
Fernandez-Garcia et al. [5]	58	Oxybuprocaine hcl 0.4%	558 ± 35	560 ± 36	3	Orbscan II
Asensio et al. [11]	26	Oxybuprocaine hcl 0.4%	559 ± 41	561 ± 42	3	Orbscan II
Rosa et al. [12]	78	Oxybuprocaine hcl 0.4%	546.76 ± 35.3	547.76 ± 36.56	5	Pentacam
Ogbuehi et al. [13]	30	Oxybuprocaine hcl 0.4%	526 ± 23	526 ± 24	10	Specular microscopy
Almubrad et al. [14]	50	Oxybuprocaine hcl 0.4%	509 ± 38	508 ± 33	5	Specular microscopy
Huang et al. [21]	53	Oxybuprocaine hcl 0.4%	551 ± 32	552 ± 35	5	Orbscan II
Mukhopadhyay et al. [22]	35	Proparacaine hcl 0.5%	554 ± 39.3	559.6 ± 40.8	1	Orbscan IIz
			546.8 ± 40	555.8 ± 38.9		Pentacam
Manassakorn and Chaidaroon [23]	19	Tetracaine hcl 0.5%	527.8 ± 32.2	530.0 ± 30.0	5	Orbscan II
Osuagwu and Ogbuehi [24]	50	Tetracaine hcl 1%	516 ± 36	515 ± 36	10	Specular microscopy
Montero et al. [25]	80	Oxybuprocaine hcl 0.4% with tetracaine hcl 0.1%	541 ± 32	541 ± 32	5	Orbscan II
